# Chinese college students’ mental health during the first three months of the COVID-19 pandemic: the protective role of family functioning

**DOI:** 10.3389/fpubh.2024.1383399

**Published:** 2024-04-25

**Authors:** Zihao Zeng, Karen Holtmaat, Irma M. Verdonck-de Leeuw, Sander L. Koole

**Affiliations:** ^1^Department of Clinical, Neuro and Developmental Psychology, Vrije Universiteit Amsterdam, Amsterdam, Netherlands; ^2^Amsterdam Public Health, Mental Health, Amsterdam, Netherlands; ^3^School of Educational Science, Hunan Normal University, Changsha, China; ^4^Cancer Center Amsterdam, Treatment and Quality of Life, Amsterdam, Netherlands; ^5^Department Otolaryngology-Head and Neck Surgery, Amsterdam UMC, Vrije Universiteit Amsterdam, Amsterdam, Netherlands

**Keywords:** COVID-19 pandemic, latent profile analysis, depression, neurasthenia, fear, obsessive-anxiety, hypochondriasis

## Abstract

**Background:**

Various psychological theories suggest that a supportive family environment protects the mental health of young adults during stressful life events. However, evidence is limited regarding the protective role of family support during a major public health crisis.

**Objective:**

To examine the role of family functioning on mental health among Chinese college students during first stage of the COVID-19 pandemic.

**Methods:**

Between January–March 2020, 1,555 college students (44% female, on average 19 years old) from five Chinese universities participated. Participants rated their family functioning on the Family APGAR Index and their mental health on the Psychological Questionnaires for Emergent Events of Public Health, measuring depression, neurasthenia, fear, obsessive-anxiety and hypochondriasis.

**Results:**

Better family functioning was associated with having fewer psychological symptoms. In addition, we identified three mental health profiles related to the severity across the psychological symptoms: Low-level, medium-level and high-level symptom clusters. Latent profile analysis showed that as family function improved, students were, respectively, 16 to 24% more likely to be in the low-level symptom group, compared to being in the medium symptom group or the high-level symptom group.

**Conclusion:**

These results support the notion that family support may act as a psychological buffer for young adults during a large-scale public health crisis like the COVID-19 pandemic.

## Introduction

The COVID-19 pandemic was a major public health emergency from 2019 to 2023, not only because of the virus’ effects on physical health, but also because the psychological threat of the pandemic and its accompanying public health measures invoked high levels of chronic life stress. This, in turn, led to global increases in mental health problems, including increased prevalence of anxiety and depression disorders (1, 2). Especially during the early stages of the pandemic, the eyes of the world were directed toward China, that the pandemic had started there. Given China’s large population and the fact that China was the epicenter of the COVID-19 pandemic, understanding its public health situation at this moment in time is of particular significance. The purpose of the present study was to investigate Chinese college students’ mental health during the first 3 months of the pandemic in China (January–March 2020) and to address the potential protective role of family functioning therein.

Life during the COVID-19 pandemic presented unique challenges for young adults worldwide, including Chinese college students. There were widespread concerns regarding the risk of contracting the virus and its potential impact on physical health (3–6). Additionally, daily routines were significantly disrupted by extensive public health measures aimed at curbing the spread of the virus, such as mask-wearing, social distancing, and the closure of public spaces like shops, schools, and restaurants. Adjusting to these changes was particularly stressful for the general population and young adults alike (7, 8). Young adults typically face elevated risks of psychological issues due to their lower levels of psychological maturity and distress tolerance (9). Furthermore, young adulthood represents a crucial developmental period characterized by rapid shifts in personality functioning (10). Persistent challenges in coping with stress during young adulthood may therefore lead to enduring vulnerabilities to mental and physical health conditions throughout the lifespan (11, 12).

Several strands of evidence highlight the heightened coping challenges faced by young adults during the COVID-19 pandemic. In a systematic review and meta-analysis involving 13,247 nursing students, Mulyadi et al. (13) found elevated rates of mental health issues, including depression (52%), fear (41%), anxiety (32%), stress (30%), and sleep disorders (27%) amidst the pandemic. Similarly, an international study encompassing over 134,000 college students across 28 countries revealed various COVID-19-related concerns among students, such as fear of virus transmission within their social circles, increased loneliness, reduced motivation, disrupted sleep patterns, and symptoms of anxiety and depression (14). Furthermore, a survey examining the mental health status of 3,881 college students during the initial 3 months of the pandemic in Guangdong, China, documented incidence rates of clinical depression and anxiety at 21 and 26%, respectively (15).

Despite the pervasive challenges posed by the COVID-19 pandemic, it appears that certain young adults were adapting effectively to their new life situation. One key resource in this regard may be the social support network of young adults, particularly their family. Family functioning encompasses the social and structural aspects of the overall family environment, including interactions and relationships within the family (16). Effective family functioning provides various psychological benefits, such as promoting secure attachment (17), enhancing a sense of belonging (18), and offering ample opportunities for social–emotional support (19). Moreover, within Chinese culture, the family holds paramount importance as one of the foundational social institutions, with traditional Chinese philosophy emphasizing harmony within the family as a fundamental virtue (20). Consequently, family functioning may exert a more pronounced influence on the mental health of Chinese adolescents (21).

In line with this perspective, a young adults’ healthy development is closely linked to strong family functioning (22–24). Children raised in well-functioning families are less prone to mental health issues compared to those from dysfunctional family environments (25). Moreover, research involving 988 adolescents (aged 11–17 years) in Monteria public schools found that better family functioning was associated with improved mental and physical health outcomes (26). Finally, a meta-analysis of 8,646 children revealed a negative association (*r* = −0.22) between family function and post-traumatic stress disorder (27), suggesting that family function can protect children’s mental health against excessive stress.

In view of the aforementioned findings, family functioning may have a protective role when people are dealing with major public health threats such as the COVID-19 pandemic. Generally speaking, in the aftermath of natural or human-made disasters, children tend to display better mental health when they are in a more (versus less) supportive family environment (28). More directly to the point, research among a sample of 135 Latinx adolescents found that better family resilience was associated with improved mental and physical health outcomes during the COVID-19 pandemic (29). A study on Puerto Rican adolescents in the U.S. also found that family financial stress had an impact on COVID-19 pandemic related mental health (30). Finally, a study of 1,254 Chinese university students in the Shanghai region (an 8-h drive to Wuhan, where the pandemic started) observed that family cohesion was negatively associated with adverse stress consequences during the third month of the COVID-19 pandemic (31).

In the present study, we sought to gain more insight into the potentially protective role of family functioning for Chinese young adults during the initial stages of the COVID-19 pandemic. Specifically, we investigated family function among a sample of more than 1,500 Chinese university students: (1) during the first 3 months of the COVID-19 pandemic; (2) in a Chinese region (Hunan) close (i.e., a 2-h drive) to the region from where COVID-19 first broke out (Wuhan); and (3) including a comprehensive and well-validated measure of mental health as our primary outcome variables. The present study thus goes beyond prior published studies (31, 32) that were conducted somewhat later in time, in regions farther removed from the epicenter of the pandemic, and did not include direct measures of mental health. In line with prior research, we hypothesized that better (rather than worse) family functioning would be positively associated with mental health. A second, more exploratory, objective of the present study was to use latent profile analysis within a regression mixture model to identify mental health subgroups within the sample of Chinese college students (33).

## Methods

### Design, participants and procedure

A cross-sectional study was conducted among students attending five different universities in China. These universities were randomly selected from Hunan Province (central China) and were all higher research universities. Participants could be included if they were (1) current university students, and (2) native Chinese speakers. All measurements were carried out with written informed consent from the universities and participants. The questionnaire was distributed to the students electronically, and all responses were anonymized to ensure confidentiality. During the COVID-19 pandemic the Hunan Agricultural University ethics committee conducted a fast-track ethical approval of studies related to the pandemic, including the present study. The study was conducted consistent with the principles of the Helsinki declaration regarding research with human subjects (34).

We used G-Power 3.1 software to identify the required sample size. To achieve a statistical power of 80%, a small effect size (0.01) and an alpha at 0.05, the required sample size was 1,199.

### Instruments

#### Sociodemographic questionnaire

We included sociodemographic variables that were found to be associated with mental health among college students in prior literature (35). We assessed sex, whether the participant was an only child, family location and monthly family income. Family location was categorized as *major city, medium city, county town* and *countryside*. Monthly family income was categorized from *below € 400* to *over € 2,500*.

#### Mental health

To evaluate participants’ mental health, we employed the original Psychological Questionnaire for Emergent Events of Public Health (PQEEPH). Originally developed during the 2003 SARS epidemic (36), this questionnaire has demonstrated strong reliability and validity in previous studies and is well-suited for assessing individuals’ psychological responses to sudden public health crises (37, 38). The PQEEPH comprises five subscales: depression (e.g., less energy than before; six items), neurasthenia (e.g., very concerned about any symptoms you may have; five items), fear (e.g., fear that you and your family may be infected; six items), obsessive-anxiety (e.g., unable to control excessive fear and nervousness; five items), and hypochondriasis (e.g., symptoms associated with a sudden public health event cause you to that suspect you that have been infected, two items). Responses to items were scored on a four-point scale ranging from 0 (“never”) to 3 (“always”). Subscale scores were calculated by summing relevant item scores and dividing by the number of items. In this study, the aggregate PQEEPH index exhibited high internal consistency, with a Cronbach’s α of 0.91. For individual subscales, Cronbach’s α values ranged from 0.67 to 0.87, indicating satisfactory reliability for scientific research purposes.

#### Family functioning

The Family APGAR index, initially developed by Smilkstein (39), underwent adaptation and translation into Chinese by Zhang (40). Subsequent research demonstrated the scale’s robust reliability and validity among Chinese college students (41, 42). Comprising five items, the scale aims to assess various facets of family functioning, encompassing adaptation, partnership, growth, affectation, and resolve. Responses are recorded on a three-point scale ranging from 0 (“never”) to 2 (“always”). In this study, the Cronbach’s α coefficient for the Family APGAR index was calculated to be 0.90, indicating high internal consistency.

### Statistical analyses

Due to the reliance on self-reported data, there exists a potential concern regarding common method bias. To address this issue, we assessed common method bias using Harman’s one-way method and confirmatory factor analysis, as recommended by Jordan and Troth (43). Furthermore, we examined the association between family functioning and mental health using Pearson correlation coefficients.

Latent profile analysis identifies cohesive latent classes or subgroups within heterogeneous data samples. To evaluate the adequacy of the latent profile model, various statistical metrics were assessed. Firstly, entropy values were scrutinized, with a threshold of ≥0.80 indicating a 90% accuracy in assigning individuals to their respective clusters, while values <0.65 suggested elevated classification errors (44). Secondly, the Bayesian Information Criterion (BIC) was utilized, with lower values indicating improved model fit as it promotes parsimony (45). Akaike’s Information Criterion (AIC) was also examined to support the chosen model. Additionally, the significance of the Lo-Mendeel-Rubin (LMR) and Bootstrap-Lo-Mendeel-Rubin (BLMR) values aided in determining the optimal number of latent classes, with these indices indicating that a solution with ‘k’ clusters significantly outperforms a ‘k – 1’ cluster model (46). Following the selection of the best-fitting model, an ANOVA was employed to assess potential differences among the mental health clusters identified in this model. In the final step of the regression mixture model, family functioning was examined in relation to the identified mental health clusters. Specifically, a logistic mixture model regression analysis was conducted to determine whether family functioning predicts membership in the clusters.

Statistical analyses were conducted using SPSS Version 27.0 (IBM, United States) and SPSS Amos software Version 26.0 (IBM, United States). Latent Profile Analysis was performed using Mplus 7.0. A significance level of *p* < 0.05 was utilized for all analyses.

## Results

### Study population

In total, questionnaires of 1,555 students were collected. Sociodemographic characteristics of the study population are provided in Table 1.

**Table 1:** Sociodemographic characteristics of sample (*N* = 1,555).

Characteristic	Category	*N*	%
Age (*M*, *SD*)	18.98 (1.28) years old		
Gender	Male	870	55.9
	Female	685	44.1
Whether only child	Only child	497	32.0
	Non-only child	1,058	68.0
Family location	Major city	122	7.8
	Medium city	301	19.4
	County town	485	31.2
	Countryside	647	41.6
Monthly family income	Below 3,000 yuan	316	20.6
	3,000–5,000 yuan	494	31.8
	5,000–8,000 yuan	345	22.2
	8,000–10,000 yuan	211	13.6
	10,000–15,000 yuan	113	7.3
	15,000–2,000 yuan	40	2.6
	Over 20,000 yuan	36	2.3

### Common method deviation test

The principal component analysis revealed that six factors had eigenvalues exceeding 1, with the variance explained by the first factor amounting to 31.64%, falling below the critical index of 40%. Furthermore, the results of the confirmatory factor analysis indicated a poor model fit, with *χ*^2^ = 9995.82, *df* = 464, CFI = 0.59, TLI = 0.57, RMSEA = 0.12, and SRMR = 0.10. Overall, these findings suggest that there was no significant common method bias present in the data.

### Descriptive statistics and correlation analysis

Table 2 displays the means, standard deviations, and intercorrelations among all study variables. Notably, family functioning exhibited negative correlations with each subscale of mental health problems.

**Table 2:** Intercorrelations among study variables.

Variables	*M*	*SD*	1	2	3	4	5
1 Depression	1.39	0.50					
2 Neurasthenia	1.40	0.47	0.64^**^				
3 Fear	1.81	0.51	0.45^**^	0.59^**^			
4 Obsessive-anxiety	1.15	0.31	0.63^**^	0.66^**^	0.41^**^		
5 Hypochondriasis	1.22	0.38	0.41^**^	0.60^**^	0.47^**^	0.65^**^	
6 Family functioning	5.86	2.50	−0.19^**^	−0.22^**^	−0.10^**^	−0.16^**^	−0.12^**^

^*^p < 0.05, ^**^p < 0.01, ^***^p < 0.001, same below.

### Effects of sociodemographic variables and family functioning on college students’ mental health

Table 3 presents the results of the regression analyses examining the influence of sociodemographic variables and family functioning on college students’ mental health. The findings reveal that family functioning significantly impacts students’ mental health. Additionally, sex and family location are associated with fear, obsessive-anxiety, and hypochondriasis. Furthermore, monthly family income demonstrates an effect on all mental health symptoms.

**Table 3:** Linear regression analysis of sociodemographic variables and family functioning on college students’ mental health.

Variables	Depression		Neurasthenia		Fear		Obsessive-Anxiety		hypochondriasis	
	*β*	*t*	*β*	*t*	*β*	*t*	*β*	*t*	*β*	*t*
Sex	−0.06	−1.25	0.05	1.17	0.33	6.83^***^	−0.16	−4.02^***^	−0.10	−2.26^*^
Only child	0.02	0.47	0.03	0.59	0.13	2.35^*^	−0.01	−0.24	0.11	2.08
Family location	−0.05	−1.93	−0.05	−1.73	−0.07	−2.65^**^	−0.04	−1.80	−0.06	−2.28^*^
Monthly family income	−0.04	−2.66^**^	−0.04	−2.66^**^	−0.03	−2.01^*^	−0.05	−3.26^**^	−0.04	−2.51^*^
family functioning	−0.18	−7.69^***^	−0.20	−8.77^***^	−0.10	−4.19^***^	−0.13	−6.21^***^	−0.10	−4.42^***^
*R* ^2^	0.04^***^		0.05^***^		0.05^***^		0.04^***^		0.02^***^	
*F*	14.02^***^		68.95^***^		16.39^***^		13.53^***^		7.29^***^	

Sex 1 indicates male, 2 indicates female. Only child 1 indicates only child 2 indicates not-only child. Family location 1 indicates major city, 2 indicates medium city, 3 indicates county town, 4 indicates countryside. Monthly family income 1 indicates below 3,000 yuan, 2 indicates 3,000–5,000 yuan, 3 indicates 5,000–8,000 yuan, 4 indicates 8,000–10,000 yuan, 5 indicates 10,000–15,000 yuan, 6 indicates 15,000–2,000 yuan, 7 indicates over 20,000 yuan.

### Mental health during the first 3 month of the pandemic: a latent profile analysis

Next, latent profile analysis was employed to explore distinct clusters in college students’ mental health symptoms, as depicted in Table 4. Entropy values for all clusters exceeded 0.8, indicating high accuracy in assigning individuals to their respective clusters. Significance was observed in the results of the LMR and BLMR tests for Models 2, 3, and 4. Notably, Models 3 and 4 exhibited lower adjusted Bayesian Information Criterion (aBIC) values, with one cluster in Model 4 comprising only 1% of the total participants. Among the tested models, Model 3 demonstrated superior values for both AIC and BIC, along with adequate entropy, suggesting its superiority in capturing the underlying structure of the data.

**Table 4:** Latent profile fit statistics for the different models of college students’ mental health symptoms.

Model	AIC	BIC	aBIC	Entropy	LMR	BLMR	Probability of clusters
1	16100.05	16164.24	16126.12				
2	13075.62	13177.26	13116.90	0.96	499.149^**^	2980.485^**^	0.87, 0.13
3	11929.24	12068.32	11985.72	0.93	532.172^*^	1138.259^*^	0.75, 0.19, 0.06
4	11151.14	11327.66	11222.83	0.93	683.983^*^	776.998^*^	0.73, 0.18, 0.08, 0.01
5	9334.34	9334.34	9421.24	0.97	52397.128	1795.886	0.08, 0.60, 0.02, 0.08, 0.22

**Figure 1:**
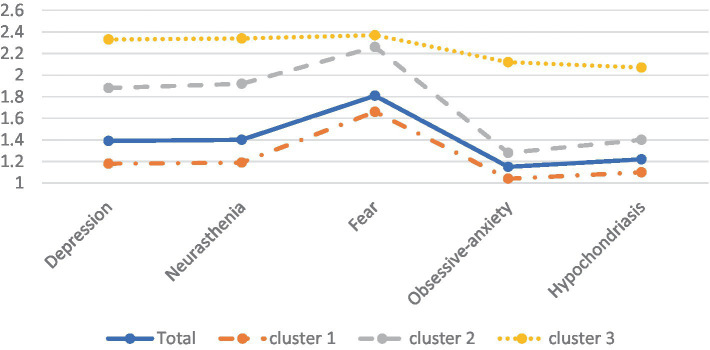
Mean symptoms for the three profiles of college students’ mental health. The *y*-axis represents PQEEPH scores.

Table 5 illustrates a comparison among three distinct clusters characterized by varying levels of mental health symptoms. The first cluster comprises individuals exhibiting low-level symptoms (*n* = 1,169, 75%), the second group manifests intermediate-level symptoms (*n* = 288, 19%), while the third group is characterized by high-level symptoms (*n* = 98, 6%), as shown in Figure 1. Single-factor ANOVA analysis revealed significant different in the mean scores of all mental health symptoms across each cluster.

**Table 5:** Mean scores of potential clusters in different mental health (standard deviations between brackets).

Project	Depression	Neurasthenia	Fear	Obsessive-anxiety	Hypochondriasis
Total	1.39 (0.50)	1.40 (0.47)	1.81 (0.51)	1.15 (0.31)	1.22 (0.38)
Low symptom cluster	1.18 (0.28)	1.19 (0.23)	1.66 (0.43)	1.04 (0.10)	1.10 (0.22)
Medium symptom cluster	1.88 (0.48)	1.92 (0.38)	2.26 (0.44)	1.28 (0.23)	1.40 (0.39)
High symptom cluster	2.33 (0.49)	2.34 (0.44)	2.37 (0.44)	2.12 (0.39)	2.07 (0.53)
*F*-value	869.27^***^	1375.99^***^	313.79^***^	2107.81^***^	590.37^***^
Multiple comparisons	1 < 2 < 3	1 < 2 < 3	1 < 2 < 3	1 < 2 < 3	1 < 2 < 3

### The influence of family functioning on the three symptom profiles: a regression mixture model

The findings from the regression mixture model are presented in Table 6. Comparing against the high-level symptom cluster as the baseline, family functioning exhibited a significant association with the low-level symptom cluster (*b* = 0.24, *SE* = 0.04, *p* < 0.001), whereas no significant association was observed with the medium-level symptom cluster (*b* = 0.08, *SE* = 0.05, *p* > 0.05). To elaborate, for each incremental increase of one point in family functioning score, there was a corresponding 24% increase in the likelihood of being categorized into the low-level symptom cluster. Conversely, when the medium-level symptom cluster served as the reference group, family functioning displayed a significant association with the low-level symptom group (*b* = 0.16, *SE* = 0.03, *p* < 0.001). In this case, with each one-point rise in family functioning score, there was a 16% rise in the probability of being classified into the low-level symptom cluster.

**Table 6:** Regression mixture model of the association between family functioning and the mental health profiles.

Predictor		Low-level cluster vs. medium-level cluster	Low-level cluster vs. high-level cluster	Medium-level cluster vs. high-level cluster
Family functioning	Intercept	0.48	1.19	0.71
	*B*	0.16	0.24	0.08
	SE	0.03	0.04	0.05
	*T*	5.17^***^	5.88^***^	1.73

## Discussion

The present study examined the association between family functioning and pandemic-related mental health among Chinese college students’ during the first 3 months of the COVID-19 pandemic. This period marked a unique phase in China’s history, characterized by unprecedented public health challenges, such largescale home quarantine for the entire population. Our findings showed that, as expected, better family functioning was associated with lower pandemic-related psychological symptoms. Additionally, higher monthly income was associated with fewer symptoms. Moreover, the results indicated that for college students, each one-point increase in family functioning score corresponded to a respective 16 and 24% greater likelihood of belonging to the low-level mental health symptom cluster compared to the medium or high-level symptom clusters. Taken together, these findings support the idea that better family functioning buffers the psychological effects of a major public health crisis, in this case among Chinese college students impacted by the stress of the COVID-19 pandemic.

The observed protective effects of family functioning in the present study align with prior findings among Chinese young adults coping with the COVID-19 pandemic (31, 32, 47) and the broader public health literature (37, 48–50). When facing a major public health crisis like the COVID-19 pandemic, people are likely to turn to their social networks for practical and emotional support. Among these networks, the family holds particular significance, perhaps especially in China, where it has traditionally occupied a central role in cultural life (20). Notably, the present study was conducted during China’s largest family-oriented holiday, the ‘Spring Festival’ (similar to the Western Christmas), so that family life was highly salient at the time of the study. Moreover, during periods of lockdown and amidst a public health crisis, the functioning of families may further gain in psychological significance. Thus, the family serves as a vital coping resource in navigating through public health emergencies.

Moreover, cultural nuances may shape how mental health symptoms are perceived and expressed. Stigma surrounding mental illness and help-seeking behaviors can vary across cultures, influencing individuals’ willingness to disclose psychological distress and seek professional support (51). Additionally, an intervention study with Iranian college athletes indicated that COVID-19 had an impact on athletic performance (52), echoing findings from a separate large-scale international study that also highlighted effects on athletes’ diet quality (53). Considering the intricate nexus between mental and physical health (54), which becomes even more pronounced during stressful events like a global health crisis, our study emphasizes the influence that physical health difficulties during the COVID-19 pandemic may have on mental well-being, highlighting the critical role of family functioning in maintaining overall health.

In developing public health policies, it is useful to know whether a population is composed of different meaningful subgroups. Our latent profile analysis revealed three distinct clusters characterized by low, middle, and high levels of symptoms. These clusters hold significance as they may represent groups with varying psychological needs. Students in the high-level symptom cluster exhibited elevated scores across all symptoms, indicating a heightened need for social and emotional support or targeted psychological counseling during public health emergencies (55). The middle-level symptom cluster, being the largest group, warrants attention as individuals within this cluster may be susceptible to transitioning into the high-level symptom cluster. Providing timely guidance and support to students in this cluster may prevent such transitions, potentially leading to a shift toward the low-symptoms cluster. However, further research is required to validate this hypothesis. As expected, fear in response to the COVID-19 pandemic emerged as the highest-scoring symptom across all clusters compared to other symptoms. Studies have indicated that nearly half of college students reported experiencing some degree of fear during the COVID-19 pandemic (56).

Amidst public health crises like the COVID-19 pandemic, a supportive family environment is particularly crucial as it serves as a psychological buffer against stress and challenges. Policymakers should consider developing and implementing policies that bolster family support systems, enhance awareness of the significance of family functioning through public health campaigns, and allocate resources to mental health services that ensure families have the necessary support to deal with stress. Educators can strengthen students’ psychological resilience by incorporating family engagement strategies and mental health education into curricula, ensuring students and their families are informed about available mental health resources. Additionally, mental health professionals should adopt family-centered therapeutic approaches (57) and develop targeted interventions that address the unique stressors of crises, focusing on strengthening family bonds and coping mechanisms. Overall, supporting students during crises requires a comprehensive approach that not only focuses on individual mental health but also values the well-being of the family as a key factor in mitigating the psychological impacts of such crises.

The current study inevitably has limitations. First, although the sample size of more than 1,500 participants was substantial, it was limited to college students from Hunan Province in China, potentially limiting the generalizability of the findings to other regions. Future research may include a broader and more varied geographic samples, longitudinal tracking, cross-cultural comparative analyses, and the adoption of mixed-methods for a comprehensive understanding. Second, the present study design was cross-sectional, precluding the testing of causal relationships between family functioning and pandemic-related psychological symptoms. It is conceivable that the relationship between family functioning and symptoms is bidirectional, with better family functioning serving as a protective factor against symptoms, and less severe symptoms facilitating better family functioning (58, 59). Future research should test the causal impact of family functioning through longitudinal or experimental designs, including the investigation of family-based interventions (60). Third, given the present study’s reliance on self-reported data, which is prone to biases like memory distortion and social desirability, future research could benefit from a multi-method approach that combines quantitative and qualitative data, including in-depth interviews or focus groups, to gain a deeper understanding of family functioning and mental health.

Despite these caveats, the present study contributes to the growing body of evidence indicating that family functioning provides psychological protection against the adverse effects of public health crises on mental health among young adults (31, 32, 61, 62). The present findings provide a unique window into the mental health of young adults in China during the first 3 months of the COVID-19 pandemic, a time when young adults were largely living in quarantine with their family. When managing largescale public health crisis, policy makers will do well to consider the protective role of family functioning in mental health.

## Data availability statement

The datasets presented in this study can be found in online repositories. The names of the repository/repositories and accession number(s) can be found at: https://osf.io/85qp3/.

## Ethics statement

The studies involving humans were approved by Hunan Agricultural University ethics committee. The studies were conducted in accordance with the local legislation and institutional requirements. The participants provided their written informed consent to participate in this study.

## Author contributions

ZZ: Conceptualization, Data curation, Funding acquisition, Writing – original draft. KH: Methodology, Writing – review & editing, Validation. IV-L: Supervision, Writing – review & editing, Validation. SK: Funding acquisition, Project administration, Supervision, Writing – review & editing.
